# Is norepinephrine more effective than other vasopressors for septic shock? A systematic review and meta-analysis

**DOI:** 10.1186/cc12159

**Published:** 2013-03-19

**Authors:** F Zhou, Z Peng, W Zhang, J Bishop, Q Song

**Affiliations:** 1 Chinese People's Liberation Army General Hospital, Beijing, China; 2 University of Pittsburgh, PA, USA; 3 University of Pittsburgh School of Medicine, PA, USA

## Introduction

Norepinephrine has been widely used in septic shock. However, its effect remains controversial. We conduct a systematic review and meta-analysis to compare the effect between norepinephrine and other vasopressors.

## Methods

The PubMed, Embase, and Cochrane Library databases from database inception until October 2012 were searched. We selected randomized controlled trials in adults with septic shock and compared norepinephrine with other vasopressors. The quality of each study included was assessed with Jadad score. After assessing for heterogeneity of treatment effect across trials using the I^2 ^statistic, we used a fixed effect model (*P *≥0.1) or random-effects model (*P <*0.1) and expressed results as the risk ratio (RR) for dichotomous outcomes or the standardized mean difference (SMD) for continuous data with 95% CI.

## Results

Eighteen trials (*n = *2,715) met inclusion criteria, which compared norepinephrine with five different vasopressors (dopamine, vasopressin, epinephrine, terlipressin and phenylephrine). The mean Jadad score was 3.11. Overall, there was no difference in mortality in the comparisons between norepinephrine and vasopressin, epinephrine, terlipressin and phenylephrine (*P >*0.05, respectively). However, norepinephrine had a trend in decreasing mortality compared with dopamine (RR, 0.84; 95% CI, 0.68 to 1.02; *P *= 0.08). There were a decreased heart rate (HR) (SMD, -2.10; 95% CI, -3.95 to -0.25; *P *= 0.03), cardiac index (SMD, -0.73; 95% CI, -1.14 to -0.03; *P *= 0.004) and an increased systemic vascular resistance index (SVRI) (SMD, 1.03; 95% CI, 0.61 to 1.45; *P <*0.0001) with the treatment of norepinephrine compared with dopamine.

## Conclusion

There is not sufficient evidence to prove that norepinephrine is superior to vasopressin, epinephrine, terlipressin and phenylephrine in terms of mortality. However, norepinephrine is associated with a decreased HR, cardiac index and an increased SVRI, and appears to have a greater effect on decreasing mortality compared with dopamine.
